# In Situ Production of Copper Oxide Nanoparticles in a Binary Molten Salt for Concentrated Solar Power Plant Applications

**DOI:** 10.3390/ma10050537

**Published:** 2017-05-19

**Authors:** Mathieu Lasfargues, Graham Stead, Muhammad Amjad, Yulong Ding, Dongsheng Wen

**Affiliations:** 1 School of Chemical and Process Engineering, University of Leeds, Leeds LS2 9JT, UK; g.stead1988@gmail.com (G.S.); pmmamj@leeds.ac.uk (M.A.); 2 School of Chemical Engineering, University of Birmingham, Birmingham B15 2TT, UK; Y.Ding@bham.ac.uk; 3 School of Aeronautic Science and Engineering, Beihang University, Beijing 100191, China

**Keywords:** in-situ production, nanoparticles, molten salts, solar energy, specific heat capacity, CSP, concentrated solar power

## Abstract

Seeding nanoparticles in molten salts has been shown recently as a promising way to improve their thermo-physical properties. The prospect of such technology is of interest to both academic and industrial sectors in order to enhance the specific heat capacity of molten salt. The latter is used in concentrated solar power plants as both heat transfer fluid and sensible storage. This work explores the feasibility of producing and dispersing nanoparticles with a novel one pot synthesis method. Using such a method, CuO nanoparticles were produced in situ via the decomposition of copper sulphate pentahydrate in a KNO_3_-NaNO_3_ binary salt. Analyses of the results suggested preferential disposition of atoms around produced nanoparticles in the molten salt. Thermal characterization of the produced nano-salt suspension indicated the dependence of the specific heat enhancement on particle morphology and distribution within the salts.

## 1. Introduction

Molten salts have been considered as a promising heat transfer fluid (HTF) replacement for synthetic oil in concentrated solar power plants (CSP) and could as well be used for thermal energy storage materials in both renewable and conventional energy systems. Currently, the most utilized heat transfer fluid in CSP application is synthetic oil such as ‘VP1 Therminol’, which is a binary mixture of biphenyl and diphenyl oxide. However, its high purchasing price, low vapor pressure, toxicity to the environment and low maximum temperature (390 °C) are pushing industrial and academic researchers to look for alternative materials. Molten salts are the solution to this challenge; in fact, a binary mixture of sodium (60 wt. %) and potassium nitrate (40 wt. %), also dubbed ‘Solar Salt’ [1], has been the most used medium for this application. However, molten salts suffer the problem of low specific heat capacities (c_p_). Introducing nanoparticles into a salt to improve its thermo-physical property is an effective solution, which has been intensively studied in the last decade [2,3,4,5,6]. However, the dispersion of nanoparticles into molten salts to form a fairly homogeneous mixture is still a highly challenging process as the materials are in solid phase at temperatures below the melting point of the salt.

To resolve the problem, Shin and Banerjee (2010) dissolved a mixture of carbonate salts into deionized water containing 1.5% by mass of SiO_2_ nanoparticles of 1–20 nm^2^. The solution was sonicated for a couple of hours and then evaporated in a petri-dish, leading to the formation of crystalline structures of coarse and fine nature. The fine structure was proposed to be responsible for the experimentally observed enhancements of specific heat by 34% and 101% for the solid and liquid phase, respectively; whilst the coarse structure gave an increase by 20–24% (solid phase) and 73–75% (liquid phase). The change in the spatial arrangement of the nanoparticles was proposed by Shin and Banerjee (2010) for the alteration of ability to store energy.

There are several hypotheses attempting to explaining the enhancement of specific heat by nanoparticles, which cannot come from the nanoparticles themselves as the latter display low specific heat capacity (i.e., typically below 1.0 J/[g*K]). Instead, the enhancement must come from the interaction between the nanoparticles and the salts, which has been partly demonstrated by Oh et al. (2005), who showed the formation of layers of aluminum atoms in liquid form at the solid interface of sapphire [7]. In a similar fashion, it is thought that the salt’s ions formed nano-layers at the interface with nanoparticles such that the atoms at this particular boundary are constrained into a semi-solid structure [8]. This formation would not behave like a typical liquid. It is further hypothesized that the observed enhancement could be due to the partial incorporation of the enthalpy of fusion as a mean to explain this rise in c_p_. Furthermore, the specific heat capacity of the nanoparticles themselves is enhanced when the overall size is reduced compared to their bulk counterparts, as described and tested by Wang et al. (2001, 2006) and Tan et al. (2009) [9,10,11]. Indeed, reducing the size of particles increases their specific surface area and the number of surface atoms exposed. The latter have far more freedom compared to atoms closer to the center, leading to a blue shift in wave number as observed by Wang et al. (2001), indicating a higher surface energy compared to bulk for Al_2_O_3_ when tested by FT-IR spectrophotometer [9]. Finally, the interfacial thermal resistance between the solid nanoparticles and the surrounding liquid could also be a mechanism for the storage of energy [12]. Whilst all of those cited mechanisms might play a part in the enhancement, the production of nano-layer seems to be the most involved player.

As summarized in Table 1, a number of publications have been made on the enhancement of specific heat of molten salts using nanoparticles [2,3,4,5,6,7,8,13,14,15,16,17,18,19,20,21,22,23]. However, most publications (Table 1—First 16 Rows) follow the same methodology of production with very little deviation [21,22]. These methodologies can be generally called ‘two-step’ methods, where nanoparticles were purchased or produced first, and then dispersed into a base salt. Indeed, whilst the dissolution of salt and the sonication of nanoparticles promote a good dispersion and homogeneity, the evaporation of water and eventual crystallization of the salt would lead to the production of heterogeneous structures (i.e., a mixture of coarse and fine structures). The energy required to evaporate the water in this production process as well as the price of nanoparticles themselves is going to render this technology far too expensive to be deemed viable.

A few other methods such as the physical dispersion of grinded powder [22] or in situ production of nanoparticles could be potential ways for scale up synthesis, as both techniques could be developed into a scalable one pot synthesis, which facilitates the industrialization of the process. Lasfargues et al. (2016) proposed a similar process whereby titanium oxysulphate was thermally degraded in a binary molten salt mixture to obtain TiO_2_ nanoparticles. In an analogous manner, but using a different substrate as well as different production methodology to the one in this paper, Yan et al. (2017) attempted the production of CuO nanoparticles via a high temperature decomposition of copper oxalate, and a moderate increase in c_p_ was reported. In this paper, a novel one pot synthesis method is performed via an in situ production of copper oxide from copper sulphate pentahydrate in a binary salt to form nano-salts. Detailed characterization of the formed products, including the preferential disposition of atoms around nanoparticles and the specific heat properties, are conducted, as below.

## 2. Methodology

### 2.1. Sample Preparation

Sodium nitrate (Sigma Aldrich, Gillingham, UK, ≥99.0%), potassium nitrate (VWR Prolabo Chemicals, Lutterworth, UK, 99.9%) and copper sulphate pentahydrate (Fluka Analytica, Gillingham, UK, ≥99.0%) were purchased and prepared through two different processes. The first method is called powder mixing (PM), which involved the grinding of the relevant powder ratios (Table 2) together in a pestle and mortar before transferring the sample into a 20-mL disposable aluminum crucible. The second method is called wet mixing (WM), in which the salt mixture was dissolved into 500 mL of deionized water before crystallization on a heating plate. The aluminum container was then inserted into a furnace at 450 °C for 30 min. After this period of time, the sample was removed and placed at room temperature to crystallize. The solid mixture was crushed in a pestle and mortar to obtain a powdered mixture, which was characterized via DSC, SEM and EDX.

#### 2.1.1. DSC

The samples (30,000–35,000 mg) were inserted into 30-µL platinum crucibles and repeatedly melted and frozen (an isothermal of 150 °C for 2 min with a dynamic ramp to 300 °C at 10 °C/min, followed by an isothermal of 2 min at 300 °C, ramping down to 150 °C at 10 °C/min, with a final isotherm of 2 min; cycle repeated three times) before carrying out the c_p_ measurements. The thermal cycle employed for this latter test was an isothermal at 250 °C for 5 min, followed by a dynamic ramp to 450 °C at 40 °C/min with another isothermal of 5 min at 450 °C. A sapphire standard (27,737 mg) was used to measure the specific heat capacity. Three samples, each tested three times, were measured in order to obtain the standard deviation used in Figures 5 and 6.

#### 2.1.2. SEM and EDX

The morphological and elemental analyses of the samples were achieved through the use of a field emission SEM (Hitachi SU8230). The samples were mounted on 12-mm aluminum stub. The coating was formed using a high-resolution sputter coater with a mixture of platinum/palladium (80/20).

## 3. Results and Discussion

**Figure 1:**
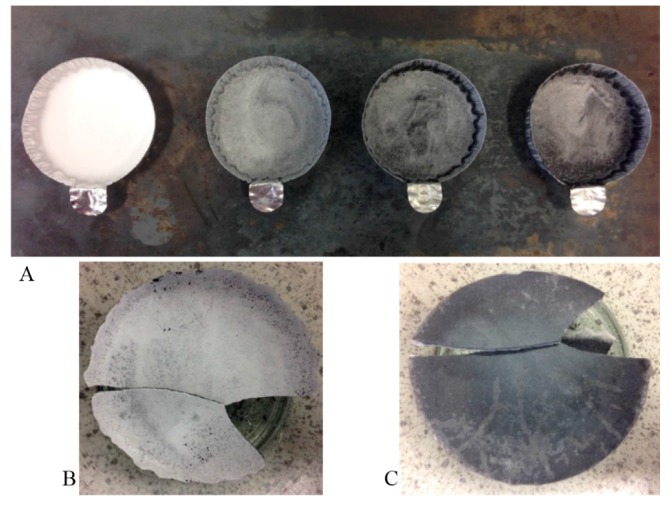
(**A**) Picture of the crucibles taken out of the furnace and left to crystallize on a sheet of stainless steel. The left crucible shows a sample of pure salt (white) (60/40 ratio–NaNO_3_/KNO_3_) whilst the next three samples (left to right) contain increasing concentrations of copper oxide calculated to be 0.32, 0.98 and 1.65 wt. %; (**B**) Top view of 0.98 wt. % CuO samples; (**C**) View of the underside of 0.98 wt. % CuO solid salt mixture.

After the removal of the aluminum holder containing the sample from the 450 °C furnace, it was cooled down to room temperature on a stainless steel sheet (Figure 1A). A visual inspection of the molten salt showed that the increase in concentration of the copper sulphate pentahydrate in the salt mixture led to darker shade of solid, due presumably to an increased concentration of copper oxide particles produced. Furthermore, it was observed that the particles in question would settle on the bottom as the underside of the salt crystal always displayed a darker color than the top part (Figure 1B,C).

Based on the stoichiometry of the reaction, the following steps take place, whereby the salt first becomes dehydrated, leading to the total removal of water, and then sulphur trioxide is released, leaving behind copper oxide (dark residue).

(1) $$Cu\left(II\right)SO_{4}.5H_{2}O+Heat\;\to Cu\left(II\right)SO_{4}+5H_{2}O$$

(2) $$Cu\left(II\right)SO_{4}+Heat\;\to CuO+SO_{3}$$

**Figure 2:**
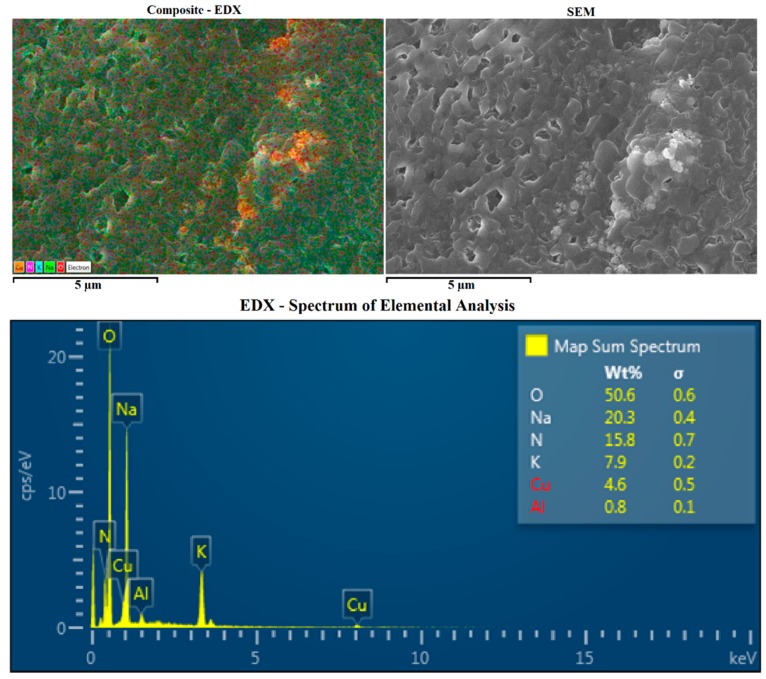
EDX Analysis of salt mixture (60% NaNO_3_ and 40% KNO_3_) with 1.65 wt. % CuO. The composite picture is an overlay of the SEM and the elemental analysis of the sample. The presence of aluminum come from the SEM stub.

It has been shown by Harris and Kalbus (1979) that the decomposition of copper sulphate pentahydrate to copper oxide requires a temperature of 1000 °C for the removal of the sulphur trioxide [24]. However, our analysis carried out by EDX did not show any sign of sulphur within the sample (Figure 2). Only nitrogen, oxygen, sodium, potassium and copper (Figure 2: EDX spectrum) were picked up, implying the formation of copper oxide only, as the sodium and potassium nitrate would have been unlikely to react with the copper sulphate pentahydrate. Indeed, from the EDX spectrum (Figure 2), we can estimate the stoichiometry of each compound with the ratio of sodium nitrate to potassium nitrate staying at 6/4, implying no reaction on its part, whilst the percentage of copper to oxygen is roughly 1 to 1.

**Figure 3:**
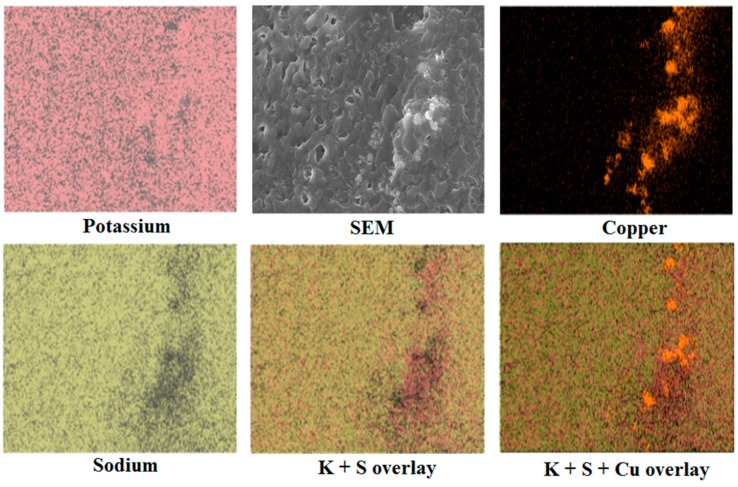
EDX mapping of potassium (red), sodium (yellow) and copper (orange) with several overlays to show the distribution of atoms.

For the EDX analysis, copper, oxygen, potassium and sodium were investigated, as previous literature [8,21] pointed out a preferential disposition of atoms around nanoparticles. Indeed, when potassium and sodium are placed on top of one another, it can be seen that the sodium atoms are less concentrated around the formed copper oxide particles (Figure 3). A similar observation has been described by Jo and Banerjee (2014) in their molecular dynamic (MD) model, and has also been shown by Lasfargues et al. (2016), with the production of titanium oxide nanoparticles using a similar technique. However, we show here experimentally that such a preferential deposition phenomenon can indeed happen. This phenomenon could be explained by the extra shell of electrons available in the potassium atoms, which shields the atoms more effectively from its nucleus, therefore allowing a greater interaction with the particles produced compared to the sodium atoms. The MD simulation of Jo and Banerjee (2014) also revealed greater adhesion forces between potassium ions and graphite compared with that of lithium [8], which further supports our observation.

**Figure 4:**
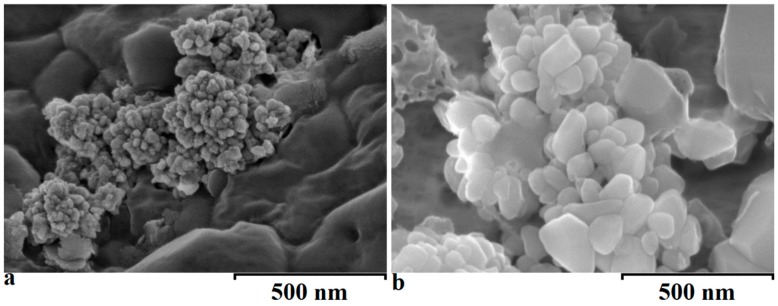
SEM images of copper oxide particles produced either through (**a**) wet mixing process or (**b**) powder mixing process. Magnification is 100.00 k.

The SEM analysis showed that the wet mixing method provided particles of a smaller size within the nanometer range, although the latter were heavily aggregated. Similarly, the powder mixing process led to the production of far larger particles of copper oxide with heterogeneous size and shape (Figure 4). Although the production process has been achieved and copper oxide nanoparticles have been successfully produced, the problem of aggregation still need to be resolved (Figure 4). The use of a dynamic process (high or low shear mixing/sonication) to reduce the agglomeration of these formed copper oxide nanoparticles in the wet mixing method could be employed. However, at these temperatures, it is likely that specific equipment would need to be built. Regarding the powder mixing process, the use of a blender rather than a pestle and mortar would provide better dispersion and homogenization of the mixture prior to the heating process.

**Figure 5:**
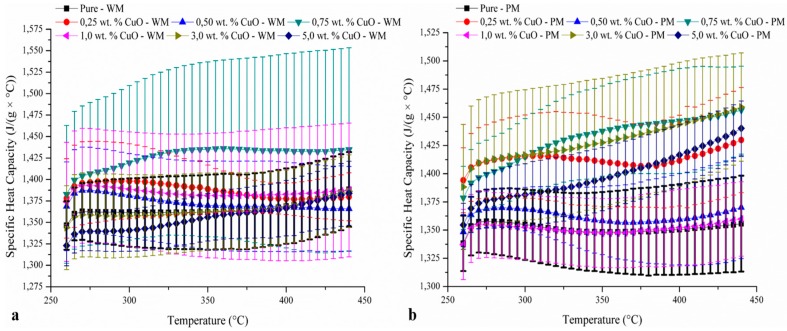
Specific heat capacity of the different ratios of salt tested using both the wet mixing (**a**) and the powder mixing (**b**) methods.

**Figure 6:**
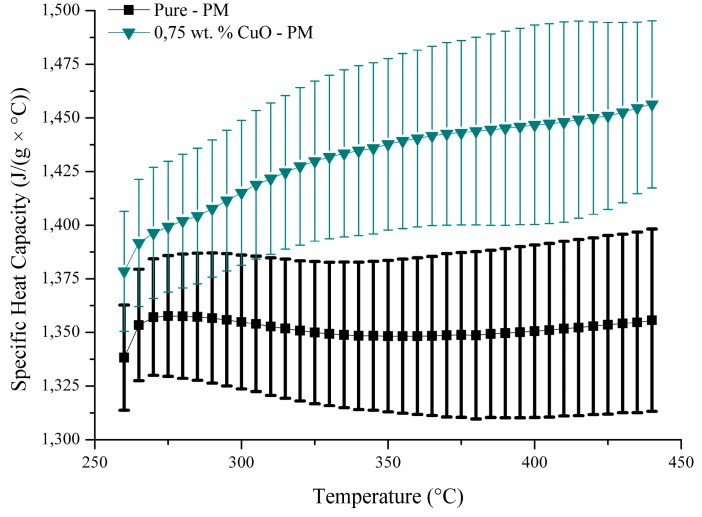
Specific heat capacity of the normal binary salt mixture against the enhanced mixture with 0.75 wt. % CuO using the powder mixing method.

The DSC measurement shows that the preparation method can affect the specific heat significantly, which is closely related to the particle morphology and distribution in the molten salt. These heavily aggregated particles impeded the specific heat capacity and did not cause any significant increase (Figure 5). It is hypothesized that for relatively well-dispersed particles, such as 0.75 wt. % CuO produced through PM (Figure 6), a good increase in the specific heat (5.6% at 320 °C to 7.4 at 440 °C-Figure 6) was observed. It is clear that the production of nanoparticles affects the c_p_ value of the molten salt and, looking only at the average c_p_ value and discarding the error bars, it can be seen that the powder method seems more effective at enhancing this property. Finding a way to better disperse the copper sulphate pentahydrate during the production process would directly impact the c_p_ value. The enhancement seen with 0.75 wt. % CuO through powder mixing is likely to be caused by the nano-layering effect, whereby ions would be constrained into a semi-solid structure at the interface with the solid CuO. It is hypothesized that the aggregation of CuO would prevent the formation of such a semi-solid structure, leading to no or little enhancement of the specific heat capacity.

Such results were in line with the literature data, which showed that the effective specific heat could be higher or lower than the base salt, depending on particle morphology. Other parameters, such as melting temperature, showed a slight decreasing trend with the increase of the copper oxide concentration, whilst the enthalpy of fusion varied only slightly (Table 3). The decrease in melting temperature could be explained by an increase in entropy due to the presence of copper oxide altering the crystal formation in the melting and crystallization processes.

## 4. Conclusions

This paper shows the potential of using feedstock copper sulphate pentahydrate to produce copper oxide nanoparticles within a binary molten salt phase of a 60% sodium nitrate and 40% potassium nitrate environment. The results showed the preferential disposition of atoms around produced nanoparticles and different levels of specific heat enhancement, depending on the particle morphology and distribution inside the molten salts.

Most of the samples did not show a significant increase in c_p_ compared to the base salt. However, when 0.75 wt. % of copper sulfate pentahydrate was added to the molten salt, a significant increase in c_p_ was achieved between 325 °C and 440 °C.

Whilst it is true that well-controlled nanoparticle production was not fully achieved for all the samples, i.e., the one pot reaction rendered heterogeneous particle sizes and the formation of aggregates. This type of process could be further refined and optimized for the economical production of nanoparticle dispersions in molten salts, promoting their applications in solar power plants as effective heat transfer fluids and/or storage materials.

## Figures and Tables

**Table 1:** First 16 Rows: Research papers using the dissolution, sonication and drying method to disperse the nanoparticles within the salt [2,3,4,5,6,7,8,13,14,15,16,17,18,19,20,21,22,23]. 17th Row: Dispersion of nanoparticles through the powder mixing method [22]. 18th Row: Production of nanoparticles within molten salt using a precursor [21].

Author	Nanoparticle	Size		Concentration	Base Fluid(s)	Measured Temperature in DSC (°C)	c_p_ Enhancement(%)	Ref.
		Diameter	Length					
Shin and Banerjee—November 2010 (IJSCS)	SiO_2_	1–20 nm	-	1.5 wt. %	Li_2_CO_3_ + K_2_CO_3_ (62:38 molar ratio)	350–550	Solid Phase: 20–42%, Liquid Phase: 73–101%	[2]
Shin and Banerjee—November 2010 (Journal of Heat Transfer)	SiO_2_	20–30 nm	-	1.0 wt. %	BaCl_2_ + NaCl+CaCl_2_+LiCl	495–555	Liquid Phase: 14.5%	[3]
Shin and Banerjee—February 2011 (IJHMT)	SiO_2_	10 nm	-	1.0 wt. %	Li_2_CO_3_ + K_2_CO_3_ (62:38 molar ratio)	525–555	Liquid Phase: 19–24%	[4]
Tiznobaik and Shin—November 2012 (IJHMT)	SiO_2_	5, 10, 30 and 60 nm	-	1.0 wt. %	Li_2_CO_3_ + K_2_CO_3_ (62:38 molar ratio)	150–550	Solid Phase: 23–28%, Liquid Phase: 22–26%	[6]
Shin and Banerjee—February 2013 (Journal of Heat Transfer)	SiO_2_	2-20 nm	-	1.5% wt. %	Li_2_CO_3_ + K_2_CO_3_ (62:38 molar ratio)	150–560	Solid Phase: 38–54%, Liquid Phase: 118–124%	[17]
Dudda and Shin—February 2013 (IJTS)	SiO_2_	5, 10, 30 and 60 nm	-	1.0 wt. %	NaNO_3_ + KNO_3_ (60:40 weight % ratio)	150–450	Solid Phase: 3–10%, Liquid Phase: 8–24%	[14]
Ho and Pan—October 2013 (IJHMT)	Al_2_O_3_	<50 nm	-	0.016 wt. %, 0.0625 wt. %, 0.125 wt. %, 0.25 wt. %, 0.5 wt. %, 1 wt. % and 2 wt. %	NaNO_3_ + KNO_3_ + NaNO_2_ (7:53:40 weight % ratio)	200–350	Liquid Phase: −5.7–+19.9%	[7]
Lu and Huang—2013 (Nanoscale Research Letters)	Al_2_O_3_	13 and 90 nm	-	0.9 vol. %, 2.7 vol. % and 4.6 vol. %	NaNO_3_ + KNO_3_ (60:40 weight % ratio)	290–340	Liquid Phase: negative values	[5]
Chieruzzi, et al. 2013 (Nanoscale Research Letters)	Al_2_O_3_, SiO_2_, SiO_2_-Al_2_O_3_, and TiO_2_	13 nm, 7 nm, 2–200 nm and 2 nm	-	0.5 wt. %, 1.0 wt. % and 1.5 wt. %	NaNO_3_ + KNO_3_ (60:40 weight % ratio)	155–295	Solid Phase: −17–+58%, Liquid Phase: −20–+23%	[15]
Liu, et al. 2013 (2nd IET Renewable Power Generation)	MWCNTs, Au	Au (5 nm and 10 nm)	-	MWCNTs (0.5 wt. %, 1 wt. % and 1.5 wt. %)	NaNO_3_ + KNO_3_ (60:40 weight % ratio)	370–420	Liquid Phase: up to +100% for MWCNTs and +220% for Au	[13]
Shin and Banerjee—February 2014 (IJHMT)	Al_2_O_3_	10 nm	-	1.0 wt. %	Li_2_CO_3_ + K_2_CO_3_ (62:38 molar ratio)	355–555	Liquid Phase: ~32%	[23]
Jo and Banerjee—May 2014 (ActaMaterialia)	Graphite	-	50 nm	0.1 wt. %	Li_2_CO_3_ + K_2_CO_3_ (74.6:25.4–62:38–34:64 molar ratios)	Solid Phase: 250 and 400, Liquid Phase: 525 and 555	Solid Phase: 28–40%, Liquid Phase: 17–57%	[8]
Seo and Shin-September 2014 (Micro and Nano Letters)	SiO_2_	60 nm	-	1.0 wt. %	LiNO_3_ + NaNO_3_ + KNO_3_ (38:15:47 molar ratio)	150–400	Solid and Liquid Phase: 13%	[18]
Andreu—Cabedo, et al. 2014 (Nanoscale Research Letters)	SiO_2_	12 nm	-	0.5 wt. %, 1.0 wt. %, 1.5 wt. %, 2.0 wt. %	NaNO_3_ + KNO_3_ (60:40 weight % ratio)	250–450	Liquid Phase: 3–25%	[2]
Jo and Banerjee—September 2015 (Journal of Heat Transfer)	MWCNTs	10–30 nm	1.5 µm	0.1 wt. %, 0.5 wt. %, 1 wt. % and 5 wt. %	Li_2_CO_3_ + K_2_CO_3_ (62:38 molar ratio)	Solid Phase: 250 and 400, Liquid Phase: 525–555	Solid Phase: 12%, Liquid Phase: 15%	[16]
Schuller, et al. 2015 (IJTS)	Al_2_O_3_	40 nm	-	0.125%, 0.25%, 0.5%, 0.75, 1%, 1.5% and 2% (nominal mass fraction)	NaNO_3_ + KNO_3_ (60:40 weight % ratio)	250–450	Liquid Phase: up to 31%	[19]
Lasfargues, et al.—June 2015 (MDPI-nanomaterials)	CuO, TiO_2_	CuO—29 nm and TiO_2_—34 nm	-	0.1 wt. %, 0.5 wt. %, 1 wt. % and 1.5 wt. %	NaNO_3_ + KNO_3_ (60:40 weight % ratio)	250–450	Liquid Phase: up to 10%	[22]
Lasfargues, et al.—May 2016 (Springer-J Nanopart Res)	TiO_2_	TiO_2_—16 nm	-	1 wt. %, 2 wt. % and 3 wt. %	NaNO_3_ + KNO_3_ (60:40 weight % ratio)	250–450	Liquid Phase: max 7.5%	[21]

**Table 2:** Weight of samples used for the production of the different concentration of copper oxide nanoparticles in molten salt (the ratio of NaNO_3_ to KNO_3_ is always 60/40).

NaNO_3_ (g)	KNO_3_ (g)	CuSO_4_.5H_2_O (wt. %)	CuSO_4_.5H_2_O (g)	Total Weight (g)	Theoretical CuO (wt. %) after Production of Nanoparticles
2.9925	1.995	0.25	0.0125	5	0.08
2.985	1.99	0.5	0.025	5	0.16
2.9775	1.985	0.75	0.0375	5	0.24
2.97	1.98	1	0.05	5	0.32
2.91	1.94	3	0.15	5	0.98
2.85	1.9	5	0.25	5	1.65

(Molar mass of CuO–79.55 g/mol, molar mass of CuSO_4_.5H_2_O–249.69 g/mol. Reaction going to completion = 79.55/249.69 × 100 = 31.86%. For 1 wt. % of CuSO_4_.5H_2_O, the theoretical CuO in wt. % is equal to the following: (31.86 × 0.05/100)/(31.86 × 0.05/100 + 2.97 + 1.98) = 0.32).

**Table 3:** Melting and enthalpy of the different tested ratios of salt + nanoparticles.

Powder Mixing (PM)	Melting Point	SD	Enthalpy of Fusion	SD	Wet Mixing (WM)	Melting Point	SD	Enthalpy of Fusion	SD
	°C		J/g			°C		J/g	
60% NaNO_3_ 40% KNO_3_	221.49	0.20	107.25	2.27	60% NaNO_3_ 40% KNO_3_	220.90	0.22	106.93	1.24
0.08 wt. % CuO	218.29	0.13	107.84	0.84	0.08 wt. % CuO	218.24	0.05	108.63	1.58
0.16 wt. % CuO	218.01	0.19	105.23	1.56	0.16 wt. % CuO	217.57	0.40	106.52	2.84
0.24 wt. % CuO	217.91	0.05	106.53	1.41	0.24 wt. % CuO	217.31	0.13	107.32	3.14
0.32 wt. % CuO	217.91	0.11	108.02	1.82	0.32 wt. % CuO	217.79	0.11	106.67	3.22
0.98 wt. % CuO	217.64	0.22	107.19	2.54	0.98 wt. % CuO	217.70	0.09	106.03	2.27
1.65 wt. % CuO	217.69	0.18	104.80	1.00	1.65 wt. % CuO	217.67	0.13	103.97	1.12
